# Electrical conduction and noise spectroscopy of sodium-alginate gold-covered ultrathin films for flexible green electronics

**DOI:** 10.1038/s41598-022-14030-2

**Published:** 2022-06-14

**Authors:** Carlo Barone, Piera Maccagnani, Franco Dinelli, Monica Bertoldo, Raffaella Capelli, Massimo Cocchi, Mirko Seri, Sergio Pagano

**Affiliations:** 1 Dipartimento di Fisica “E.R. Caianiello”, Università degli Studi di Salerno, Via Giovanni Paolo II 132, 84084 Fisciano, SA Italy; 2 CNR-SPIN Salerno, c/o Università degli Studi di Salerno, 84084 Fisciano, SA Italy; 3 INFN Gruppo Collegato di Salerno, c/o Università degli Studi di Salerno, 84084 Fisciano, SA Italy; 4 CNR-Istituto per la Microelettronica e Microsistemi, Via P. Gobetti 101, 40129 Bologna, Italy; 5 CNR-Istituto Nazionale di Ottica, Via G. Moruzzi 1, 56124 Pisa, Italy; 6 Dipartimento di Scienze Chimiche, Farmaceutiche ed Agrarie, Università degli Studi di Ferrara, Via L. Borsari 46, 44121 Ferrara, Italy; 7 Istituto per la Sintesi Organica e la Fotoreattività, Consiglio Nazionale delle Ricerche, Via P. Gobetti 101, 40129 Bologna, Italy; 8 Dipartimento di Ingegneria E. Ferrari, Università di Modena e Reggio Emilia, 41125 Modena, Italy; 9 CNR-Istituto Officina dei Materiali, S.S. 14, km 163.5 in Area Science Park, 34012 Trieste, Italy; 10 Department of Physics, University of Johannesburg, P.O. Box 524, Auckland Park, 2006 South Africa; 11 CNR-Istituto per lo Studio dei Materiali Nanostrutturati (ISMN), Via Piero Gobetti 101, 40129 Bologna, Italy

**Keywords:** Materials for devices, Polymers, Surfaces, interfaces and thin films

## Abstract

Green electronics is an emerging topic that requires the exploration of new methodologies for the integration of green components into electronic devices. Therefore, the development of alternative and eco-friendly raw materials, biocompatible and biodegradable, is of great importance. Among these, sodium-alginate is a natural biopolymer extracted from marine algae having a great potential in terms of transparency, flexibility, and conductivity, when functionalized with a thin gold (Au) layer. The electrical transport of these flexible and conducting substrates has been studied, by DC measurements, from 300 to 10 K, to understand the interplay between the organic substrate and the metallic layer. The results were compared to reference bilayers based on polymethyl-methacrylate, a well-known polymer used in electronics. In addition, a detailed investigation of the electric noise properties was also performed. This analysis allows to study the effect of charge carriers fluctuations, providing important information to quantify the minimum metallic thickness required for electronic applications. In particular, the typical noise behavior of metallic compounds was observed in samples covered with 5 nm of Au, while noise levels related to a non-metallic conduction were found for a thickness of 4.5 nm, despite of the relatively good DC conductance of the bilayer.

## Introduction

The development of flexible electronic devices has received much attention in the past decade, because they are expected to have a large impact in electrical and electronic equipment (EEE), which has become an essential part of our everyday life. After its use, EEE is disposed, generating large e-waste of hazardous but valuable materials. In 2019, the World generated 53,6 million metric tons (Mt) of e-waste, and only 17.4% of this e-waste was collected and recycled^1^. Present recycling technologies are mainly based on smelter and chemical technologies. The operation of a smelter is highly energy-intensive, while the commonly used mineral acids in chemical recycling techniques pose serious environmental risks for workers as well as for the air quality and water streams^2,3^.

The use of biodegradable and easily recyclable materials in the framework of green electronics could greatly reduce the environmental impact of e-waste. In fact, these materials offer the opportunity of a more environmentally sustainable recycling route^2,4^, as well as a safe management of single-use disposable devices such as sensors. According to European Standard EN13432, a biodegradable material is the one that can be converted by at least 90% into harmless components, such as water, carbon dioxide, and biomass, through the action of fungi or microorganisms within 6 months. Currently, the use of biodegradable materials represents an opportunity in several fields, where they can be used as substrates, inter- or active- layers and electrodes. To date, the most studied and promising biodegradable materials are cellulose derivatives^5,6^, chitin/chitosan^7–9^, and silk fibroin^10–12^.

Recently, we have started employing sodium-alginate (SA), a natural biodegradable polymer derived from brown algae^13^. SA is water soluble and easy to manipulate, so that flat and transparent foils can easily be fabricated with an environmentally friendly process^14^. With the aim of using SA for the fabrication of innovative substrates to produce green devices for light and energy (i.e. organic photodiodes (OPD), organic light emitting diodes (OLEDs), polymer solar cells (PSCs), etc.), we have deposited a thin Au layer on top of a SA film obtaining a conductive bilayer, whose electrical properties need to be studied in order to tailor the substrates to the final application. This platform has been successfully integrated in working OLEDs^4^, demonstrating that it can substitute the conventional one made of glass/ITO bilayers. Very thin metallic layers are however mandatory to preserve the substrate transparency in optoelectronic applications. Therefore, in the fabrication of a disposable EEE the amount of the metallic part in the bilayer should be minimal, while sustaining a proper current transport with a high electrical conductance. Au represents a good choice for its good growth and connectivity properties but also for its resistance to chemical degradation, providing a good conductance even at a very low thickness.

Gold nanolayers ranging from 4.5 to 24 nm sputtered on SA have already been investigated by the authors, founding a sharp change (of several order of magnitude) in the resistance as a function of Au thickness, at about 4–5 nm^15,16^. This value is close to electrical percolation thickness for Au on glass in the case of Volmer-Weber growth mode (VWGM)^17^. The temperature dependence of the resistance was found to be different for gold nanolayers with thickness above and below 5 nm^16^, and the transition between the two different regimes was explained in terms of loss of the percolation paths above the critical thickness, with corresponding change in the conductance mechanism from a classic metallic to a fluctuation-induced tunneling through gold clusters embedded in the sodium-alginate film. These results have been further investigated in this paper by noise spectroscopy, using conventional substrates as reference and by studying the conduction mechanism at the threshold of metallic/non-metallic conduction, which occurs for a gold thickness of 4.5 nm. It’s well-known that the Au film growth process proceeds, in its first stages, with the formation of nanoparticles only partially connected^18^. The interconnections between the nanometric domains build up as the Au thickness increases, until a full coverage of the surface is obtained^15^. Indeed, the use of an organic substrate instead of a rigid inorganic one (as glass or mica used in Ref.^19^), may result in a different growth mechanism. Furthermore, it is obvious that the interface of the nanostructured gold layer may strongly be affected by the presence of the organic polymer. The role of such an interface on the electron transport mechanism and in formation of the electrical percolation network is not known, and it can lead to substrates with relatively high conductance but characterized by unstable conduction paths that may introduce large electric noise in real devices. The presence of fluctuating current paths can be readily evidenced performing a noise spectroscopy analysis, which allows to determine the Au layer thickness that guarantees both good electrical conductance and low noise level in these innovative conducting substrates.

In this work, we have analyzed in detail the electrical transport in SA/Au substrates with Au thicknesses of few nanometers. It is very important to stress that, when investigating ultrathin films, a high conductance cannot be considered a sufficient property. Indeed, the Au film growth process on polymeric substrates, such as SA, proceeds in its first stages with the formation of nanoparticles only partially connected. The interconnections between the nanometric domains form for increasing Au thickness, until a full coverage of the surface is obtained^15^. This network morphology can lead to substrates with relatively high conductance but characterized by unstable conduction paths that may introduce large electric noise in real devices. The presence of fluctuating current paths can be readily evidenced performing a noise spectroscopy analysis, which allows determining the Au layer thickness that guarantees both a good electrical conductance and a low noise level.

## Results and discussion

### Electric transport and noise properties near the non-metallic threshold

As already mentioned, commercially available biopolymers, even if widely used and studied in various fields ranging from packaging to medicine, are poorly investigated in electronics and in particular as innovative green substrates. In this respect, a comparison of SA with a traditional material such as polymethyl-methacrylate (PMMA), whose properties and applications in electronic devices are well-known, can provide interesting information.

**Figure 1:**
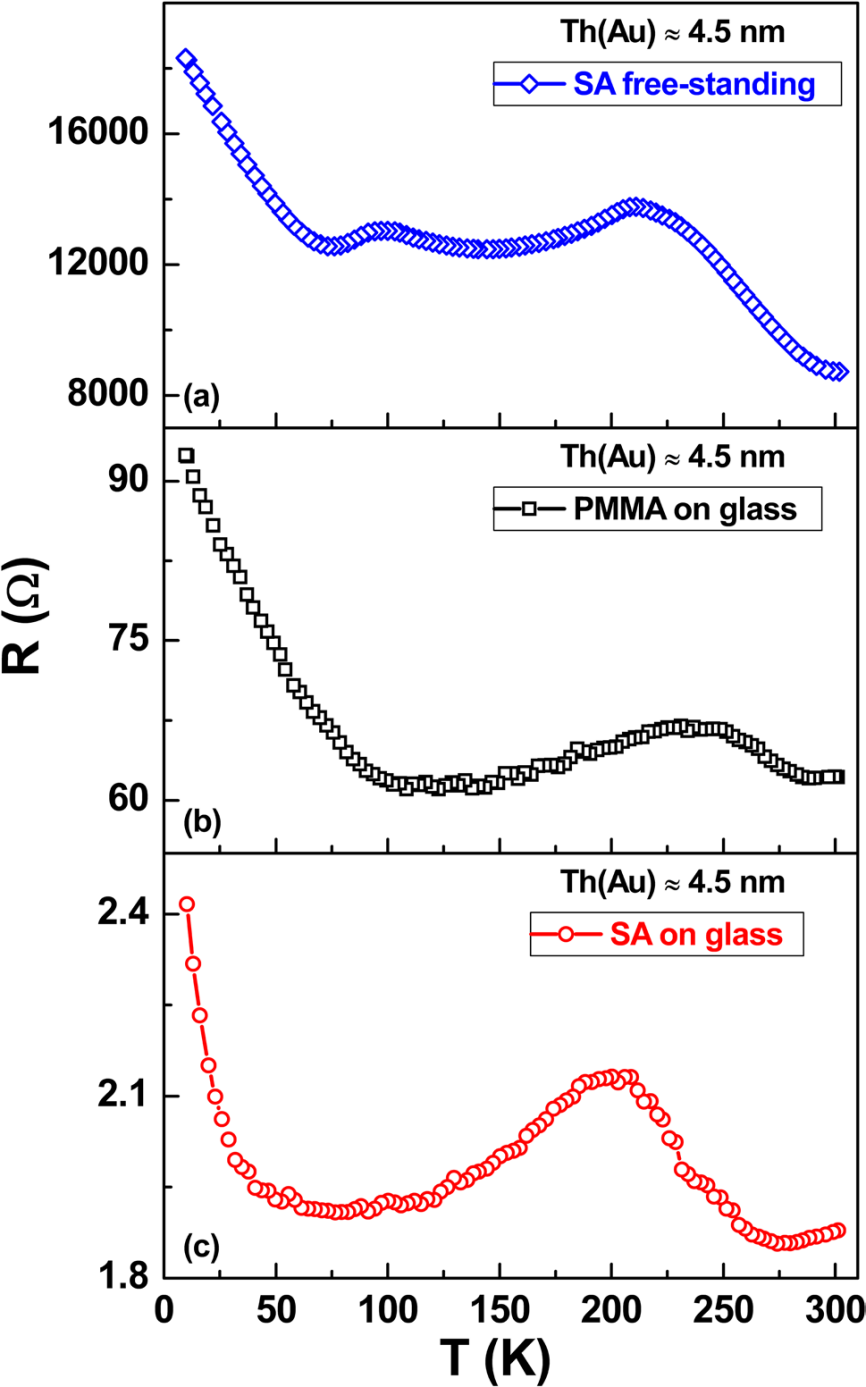
Resistance versus temperature plots of non-metallic films. The data refer to three different investigated samples covered with a 4.5 nm-thick Au layer: (**a**) SA free-standing film (blue diamonds), (**b**) PMMA deposited on glass (black squares), (**c**) SA deposited on glass (red circles).

Figure 1 shows the temperature dependence of the measured resistance *R*(*T*) for SA and PMMA films covered with a 4.5 nm-thick Au layer, representing the previously studied threshold between a non-metallic (below) and a metallic (above) behavior^16^. Both for a free-standing SA film (Fig. 1a) and SA or PMMA films spin-coated on glass (Fig. 1b,c), similar *R*(*T*) curves are observed in the whole range from 300 down to 10 K. More in details, a resistance increase by lowering the temperature is always found below 100 K, while an evident peak, more pronounced for SA films, occurs in a region around 200 K. At 300 K, a rough estimation of the gold layer sheet resistance, made on the unpatterned samples deposited on glass, gives values of ~ 37 Ω/sq for PMMA and of ~ 19 Ω/sq for SA, which are in a good agreement with those of gold nanostructures sputtered on glass^20^ and on other transparent polymers^21^.

These results confirm the general framework of the transport mechanisms already reported in literature, described in terms of a fluctuation-induced tunneling process at low temperatures^22^ and of a conductive region expansion from 300 to 200 K^16^. However, whether the few differences found in the *R*(*T*) curves are related to the role played by the presence of water, whose effect could be different depending on the polymeric matrix considered (PMMA or SA), is the interesting question to unravel. Therefore, in order to extract more information on the conduction mechanisms in working conditions, a more sensitive investigation has been performed by studying the charge carriers fluctuations, using the well-known electric noise spectroscopy technique.

In this type of noise characterizations, the main information is essentially given by the voltage-spectral density function *S*_*V*_ and, more in details, by analyzing its amplitude frequency dependence. For the samples here investigated, the best fitting procedure of the spectral traces can be obtained by using a generic expression in the form of

1 $$ S_{V} (f) = \frac{K}{{f^{\gamma } }} + S_{0} $$

**Figure 2:**
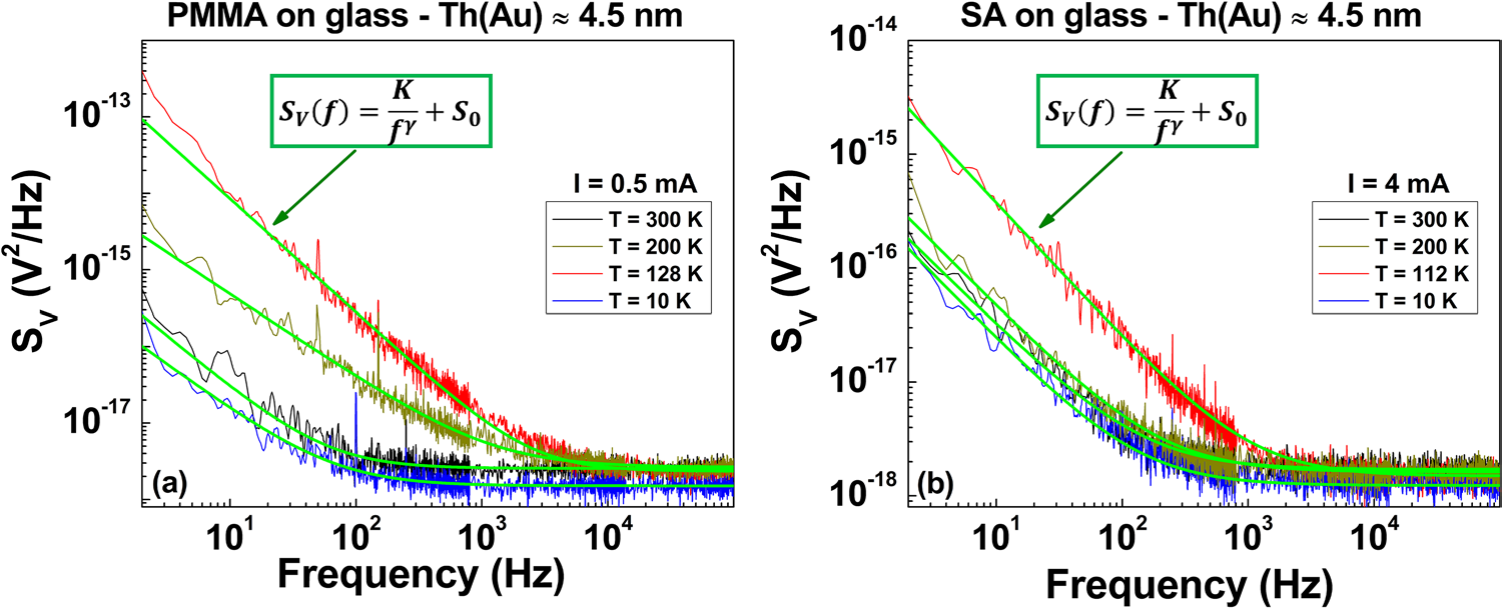
Voltage-noise spectra. The frequency dependence of *S*_*V*_, at fixed bias current values, is shown for PMMA (**a**) and for SA (**b**) films deposited on glass and covered with a 4.5 nm-thick Au layer. The green solid lines are the best fitting curves obtained by using Eq. (1).

**Figure 3:**
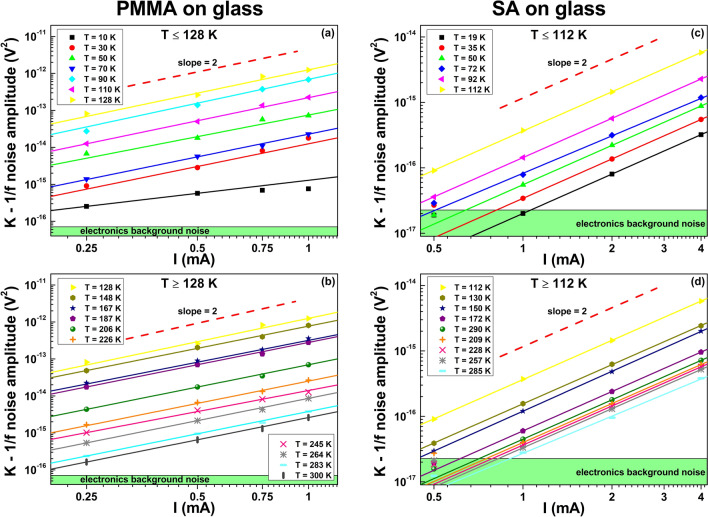
Current dependence of the 1/f noise component. The amplitude *K* of the 1/f noise is shown as a function of the applied bias current for PMMA (**a**), (**b**) and for SA (**c**), (**d**) non-metallic films. A typical quadratic behavior is always observed both below (upper panels) and above (lower panels) the temperatures at which a resistance minimum occurs for the two different investigated systems.

Here, *γ* is the noise frequency exponent, *S*_0_ is a frequency-independent term, while *K* is the noise amplitude coefficient whose study as a function of external parameters, such as temperature and bias current, allows establishing correlations and relationships with physical properties of the system involved^23,24^. The green curves in Fig. 2 show a good agreement between Eq. (1) and the experimental noise spectra, both for PMMA (left panel) and SA (right panel) Au coated films in the whole temperature range. As result of the data analysis, varying the temperature from 300 to 10 K, the exponent γ ranges in the interval between 1.2 and 1.4 on both the investigated substrates. This suggests a small number *N* of active fluctuators as responsible for the noise mechanisms^23,25,26^. In fact, a large number of Lorentzian fluctuators (*N* → ∞) would generate a pure 1/f noise component with γ values ranging from 0.8 to 1.22^3,27,28^. The constant term *S*_0_ is the “white-noise” component that essentially consists in the Johnson thermal noise (4*k*_*B*_*TR*) added to a background contribution. Due to the small resistance values measured both for PMMA and for SA films, *S*_0_ corresponds to the voltage-spectral density of the experimental setup electronic chain, being ~ 1 × 10^−18^ V^2^/Hz. The noise amplitude coefficient *K*, moreover, can be studied as a function of the applied bias current *I*, always revealing a quadratic behavior in the whole tested temperature range, as shown in Fig. 3. This is the expected standard behavior when the noise processes are originated by resistivity fluctuations in a random resistance network^28,29^.

**Figure 4:**
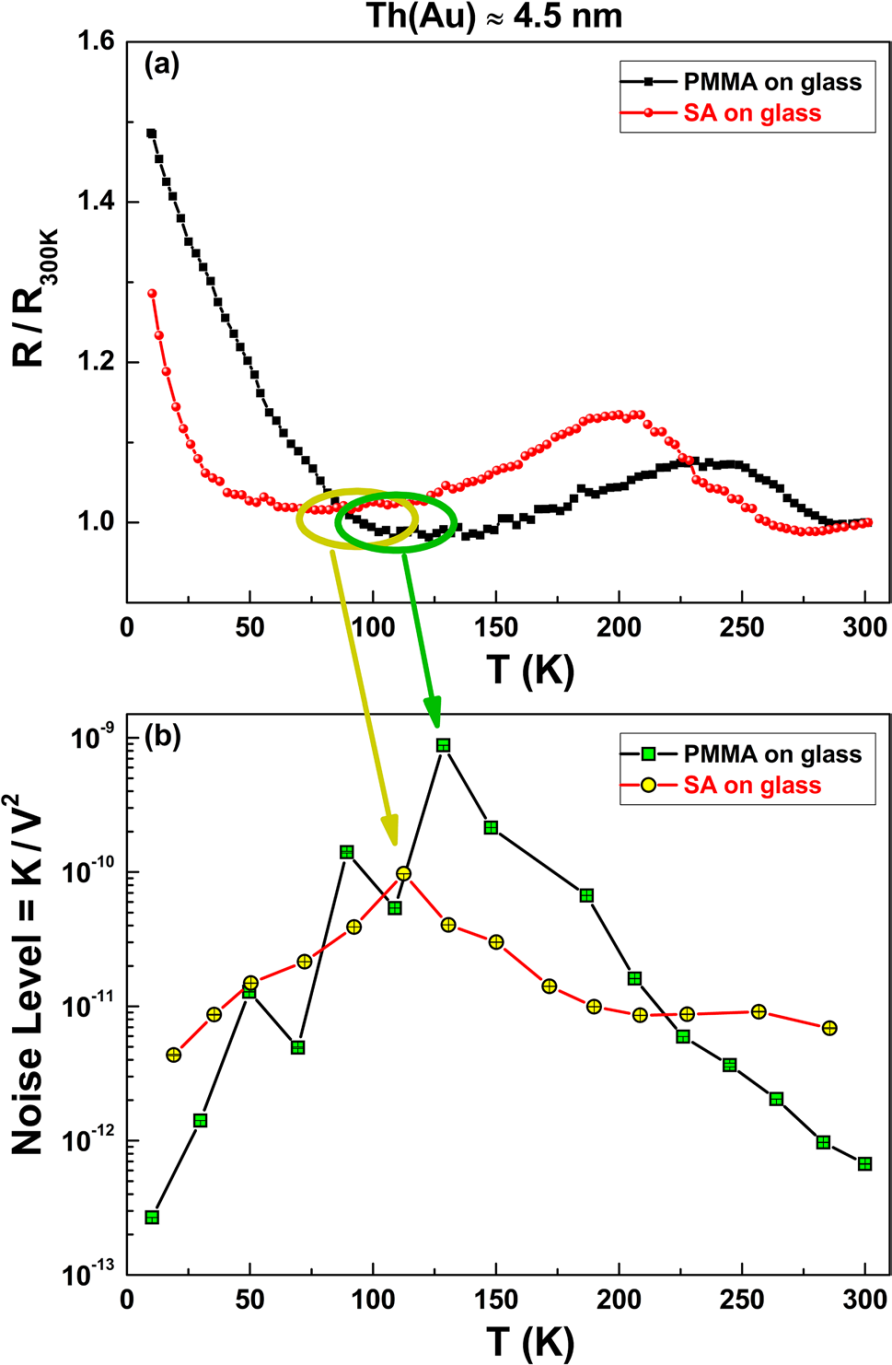
Comparison between DC and AC properties of non-metallic samples. The temperature dependencies of the normalized resistance *R*/*R*_300*K*_ (**a**) and of the Noise Level *NL* (**b**), as evaluated from Eq. (2), are shown for PMMA (squares) and SA (circles) films. The *NL* peaks, corresponding to the different resistance minima observed, are evidenced with green and yellow arrows for PMMA and SA, respectively.

Starting from the quadratic current dependence of *K*, it is straightforward to evaluate the Noise Level (*NL*) of Ohmic systems as^23^

2 $$ NL = \frac{K}{{V^{2} }} = \frac{K}{{R^{2} I^{2} }}, $$

being *V* the measured DC voltage. As evidenced in Fig. 4, a clear *NL* peak occurs in the temperature region where an upturn of the resistance is observed. This happens around 128 K for PMMA (green square) and around 112 K for SA (yellow circle). The presence of a peak in the noise level amplitude is usually associated to a change in the electric transport mechanisms^30–33^.

**Figure 5:**
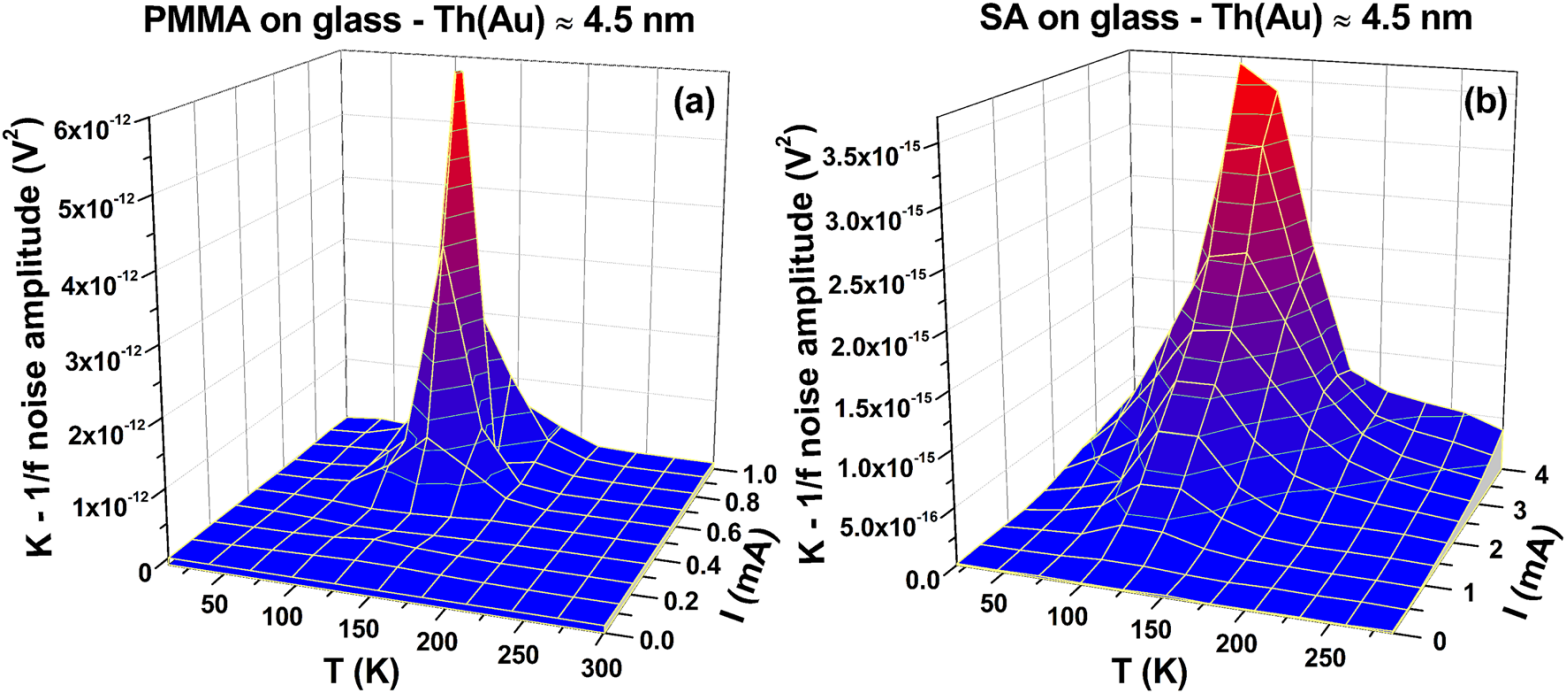
Noise properties of non-metallic samples. The amplitude *K* of the 1/f noise component is shown as a function of temperature and of bias current, in a three-dimensional plot, for PMMA (**a**) and for SA (**b**) films deposited on glass and covered with a 4.5 nm-thick Au layer.

The transition to a typical non-metallic transport is confirmed by the increase of the normalized resistance *R*/*R*_300*K*_ below 100 K for Au film thickness ≤ 4.5 nm. A comparison between the behavior on PMMA and SA shows minor differences indicating a minor role of the substrate composition on the Au growth. The ultrathin film can be regarded as a network of discontinuous metal regions instead of a continuous layer^15^, whose morphology does not depend on PMMA or SA and results in the specific non-metallic features observed. From a technological point of view, the results of fluctuations spectroscopy give the indication of low *NL* values in the low-temperature region, as expected because of the low charge carriers mobility. Moreover, similar low *NL* values are observed even at high temperatures, in the range of a typical device use. This last feature, very interesting for the development of room-temperature applications, is clearly evident in Fig. 5 three-dimensional graphs of the amplitude parameter *K*, both for PMMA (left panel) and SA (right panel). Notice that, while the general behavior of PMMA and SA spin-coated films is similar, the effect of the fluctuation processes on the room temperature electric noise is lower on SA than PMMA, thanks to the peak of *NL* shifted toward a lower temperature value in SA (see Figs. 4 and 5, for details). This results in another advantage of SA over PMMA, in addition to the flexibility and the non-fossil-oil origin.

### Electric transport and noise properties above the metallic threshold

**Figure 6:**
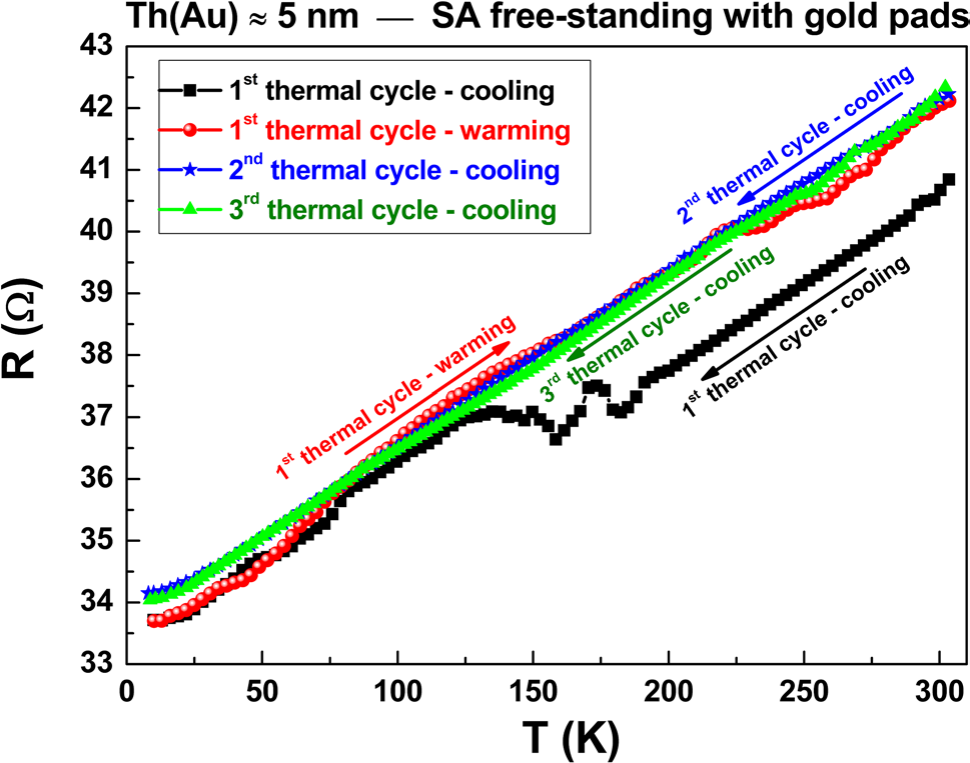
Resistance versus temperature plots of metallic films. The temperature dependence of the resistance *R* is shown for SA free-standing films covered with a 5 nm-thick Au layer. The results, obtained on samples electrically contacted with Au pads, are reported for subsequent thermal cycles.

The evidence of a *NL* peak, observed in the presence of a non-metallic conduction, completely disappears when the electric transport mechanisms change. In particular, increasing the thickness of the sputtered Au above 4.5 nm, a more metallic behavior is recovered as shown in Fig. 6 for a free-standing SA film covered with a 5 nm-thick Au layer. After a first thermal cycle (black squares, acquired in cooling mode) the polymeric matrix settles (red circles, acquired in warming mode), becoming more stable during the second thermal cycle (blue stars, acquired in cooling mode) and for all the subsequent thermal cyclic tests performed (green triangles, acquired in cooling mode). It is evident that no hysteretic effect occurs, as observed in the whole set of compounds investigated. It is important to underline that, using Au pads (60 nm-thick, deposited onto the sample surface) for the electrical connections, the measurements are usually characterized by strong stability and repeatability. Signs of instability and non-repeatability are, instead, visible in absence of Au pads, as shown in Supplementary Fig. S1 where experimental data taken on Au sputtered SA films with (red diamonds) and without (black circle) Au pads are compared.

**Figure 7:**
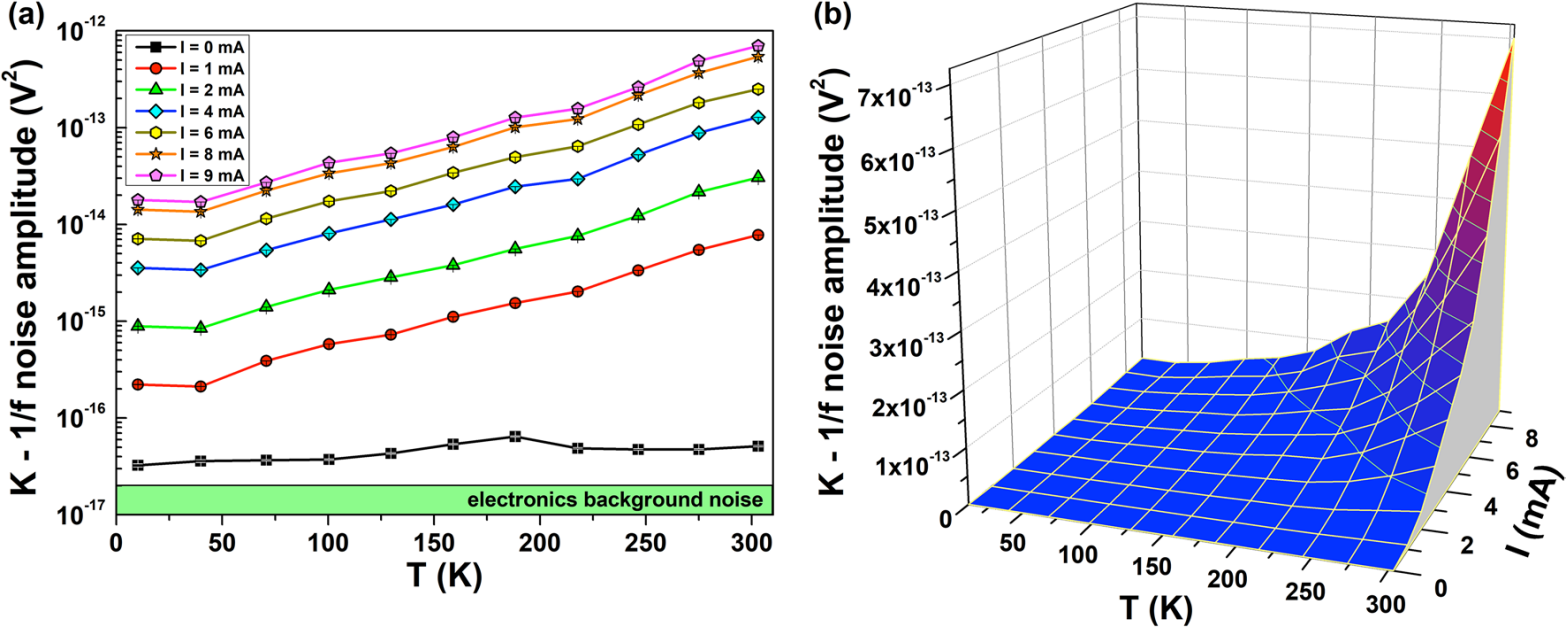
Noise properties of metallic samples. The amplitude *K* of the 1/f noise component of SA free-standing films covered with a 5 nm-thick Au layer is shown: (**a**) as a function of temperature in a two-dimensional plot for different bias current values; (**b**) as a function of temperature and of bias current in a three-dimensional plot.

Therefore, it is clear that a well-defined geometry of the contact pads allows an accurate evaluation of the intrinsic noise, as evidenced in Fig. 7 for a typical metallized SA free-standing film (Au thickness of 5 nm). More in details, the 1/f noise amplitude shows a decrease by lowering the temperature (see the two-dimensional plot of Fig. 7a). The reduction of *K* is usually expected in the case of metals, together with a quadratic current dependence of the 1/f noise component^23,27^, as shown in the three-dimensional plot of Fig. 7b, which can be attributed to random resistance fluctuations.

**Figure 8:**
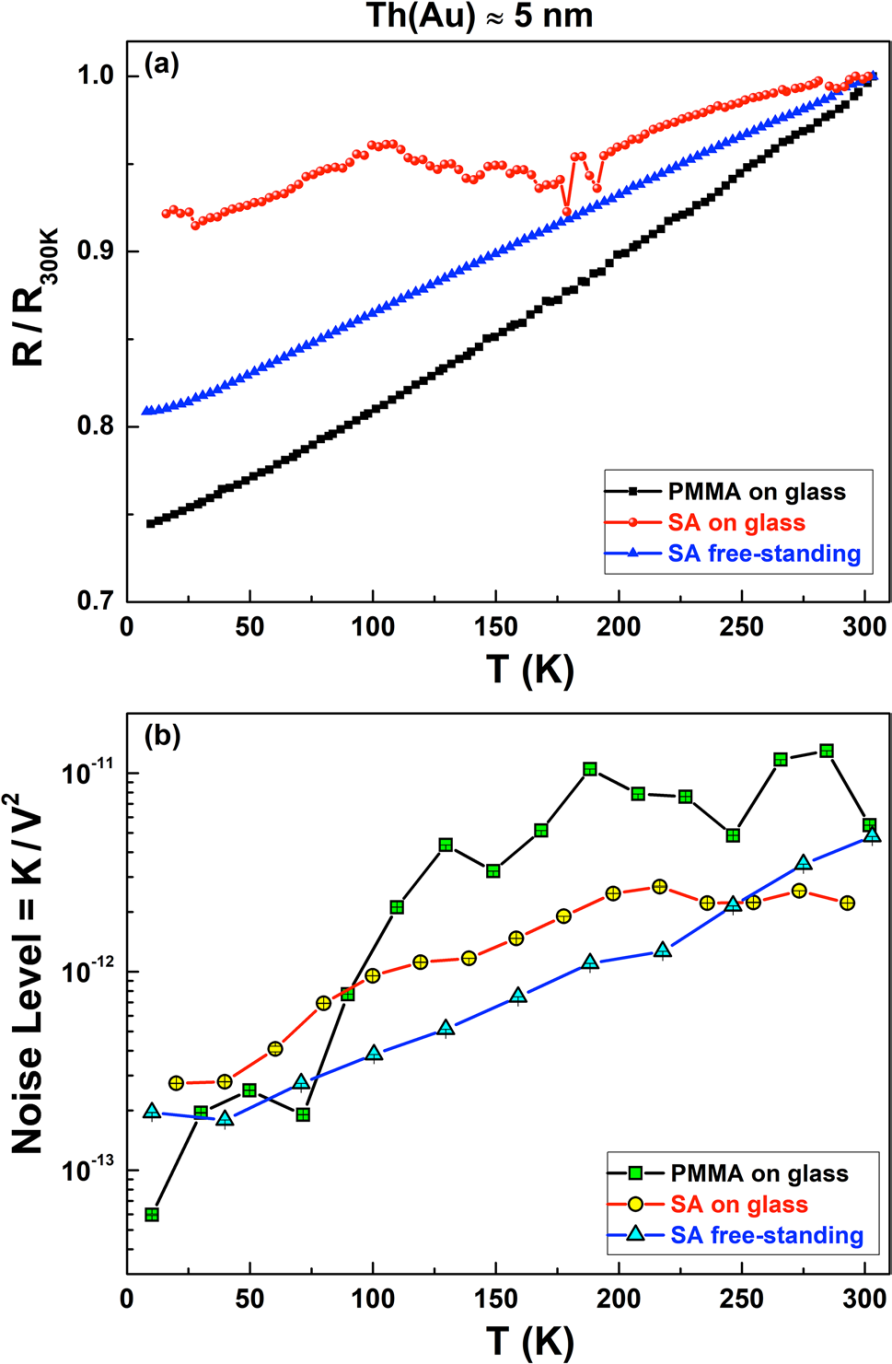
Comparison between DC and AC properties of metallic samples. The temperature dependencies of the normalized resistance R/R_300*K*_ (**a**) and of the Noise Level NL (**b**) are shown for PMMA on glass (squares), SA on glass (circles), and SA free-standing (triangles) films. The typical characteristics of metallic compounds are observed, especially regarding the very low noise measured in the whole investigated temperature range.

From the literature it is known that this type of fluctuation processes, for metal or bad-metal compounds, is characterized by a noise level reduction for decreasing temperature^34,35^. This behavior is shown in Fig. 8 for all the PMMA and SA samples covered with a 5 nm-thick Au layer, both for free-standing and spin-coated films, whose typical metallic conduction is verified in Fig. 8a in terms of the normalized resistance *R*/*R*_300*K*_. In particular, Fig. 8b clearly evidences a monotonic decrease of *NL* moving from 300 to 10 K. One possible explanation for the observed noise temperature dependence can be found in a theoretical model which ascribes the origin of resistance fluctuations to vacancy and interstitial diffusion^27,36^, as already reported for granular and polycrystalline systems^37,38^. This finding gives an indication that above a certain Au layer thickness, here identified in 4.5 nm, the conducting regions forming the ultrathin films are more uniformly distributed and interconnected, despite the possible presence of structural defects at the points of their closest distance.

From the point of view of applications, also in the case of metallic films as already discussed for non-metallic samples, it is important to stress that the intrinsic noise level is very low and has a weak dependence on the type of substrate used for the device fabrication. As a matter of fact, the free-standing foils seems to generally have a lower *NL* value compared to the compounds on glass, resulting very promising in the development of a flexible “*green electronics*”.

## Conclusions

The realization of green electronics may only start from the use of biopolymers, obtained from renewable resources, as innovative substrates. To this end, substrates made of sodium-alginate, a natural biopolymer extracted from marine algae, covered with an ultrathin gold layer have been investigated from the electrical conduction point of view. In particular, a comparative study with polymethyl-methacrylate has been carried out on films spin-coated on glass. The gold layer thickness has been kept to a minimum value in order to reduce the amount of expensive and non-biodegradable material employed while preserving good electrical conduction properties, although a possible recovery of the metals has been recently demonstrated for SA-based devices. A range between 4.5 and 5 nm has been identified as the region where the non-metallic and metallic transition occurs for both polymers. In addition, measurements have been performed on sodium-alginate free-standing films, highlighting its high potential for applications in flexible green electronics.

Measurements of DC resistivity, performed cycling the temperature from 300 to 10 K, show that samples reach stability in the electrical conduction after one thermal cycle. Moreover, important information has been obtained by measuring the noise level amplitude, which is very low and comparable with metallic materials currently employed in common electronics for samples with a 5 nm-thick gold layer. For a thickness equal 4.5 nm, the noise level amplitude is two orders of magnitude larger, indicating a completely different conduction mechanism, dominated by a random network of interconnected gold islands. This is important to define the minimum thickness of metallic layer to be used for the preparation of flexible transparent electrodes suitable for a new generation of green electronic devices.

## Methods

### Samples preparation

The SA solution (2% conc.) for spin-coating was prepared by solubilizing the appropriated amount of SA (Farmalabor Srl, Canosa di Puglia (BT), Italy) in ultrapure water under stirring at room temperature for several hours. The solution was left to stand at least overnight to allow bubbles escaping.

SA foils have been obtained casting a 4% water solution of pharmaceutical grade product (Farmalabor Srl, Canosa di Puglia (BT), Italy), into polystyrene Petri dishes^15^. Evaporation of the excess water is performed in clean room, under controlled environmental conditions (humidity ~ 40%, and temperature ~ 23 °C). On free-standing samples, Au is sputtered in the central part of a rectangular SA stripe (2.5 × 1.5 cm^2^), through a metal mask, using a MRC 8622 RF system. In order to have a fine control of Au thickness, the deposition processes were performed at 20 W. A second Au deposition (60 nm thick), is executed for the fabrication of four pads, which guarantee good and stable Ohmic contact during the long electrical measurements.

On glass substrates (6 mm by side), SA and PMMA films were deposited by spin-coating in air. In detail, the SA solution (4% w/w) was spin-coated at 2000 rpm for 60 s (thickness of ~ 400 nm) and subsequently annealed in air at 80 °C for 5 min, while PMMA was spin-coated at 5000 rpm for 60 s (thickness of ~ 350 nm) and subsequently annealed in air at 110 °C for 3 h. The thickness of the spin-coated films was measured by a profilometer (KLATencor, P-6). For these samples the thin Au film is sputtered blanket on top of SA, applying the same deposition conditions used for free standing.

### Structural and morphological characterizations

Atomic force microscopy (AFM) was performed using a hybrid system made of a commercial head (SMENA, NT-MDT), home-built electronics and a digital lock-in amplifier (Zurich HF2LI). The setup was operated in intermittent contact mode (ICM). The cantilevers employed are commercially available from MikroMasch (HQ:NSC35). The imaging size of 2 × 2 µm^2^ has been chosen in order to observe the fine texture and to provide a sufficiently wide view.

**Figure 9:**
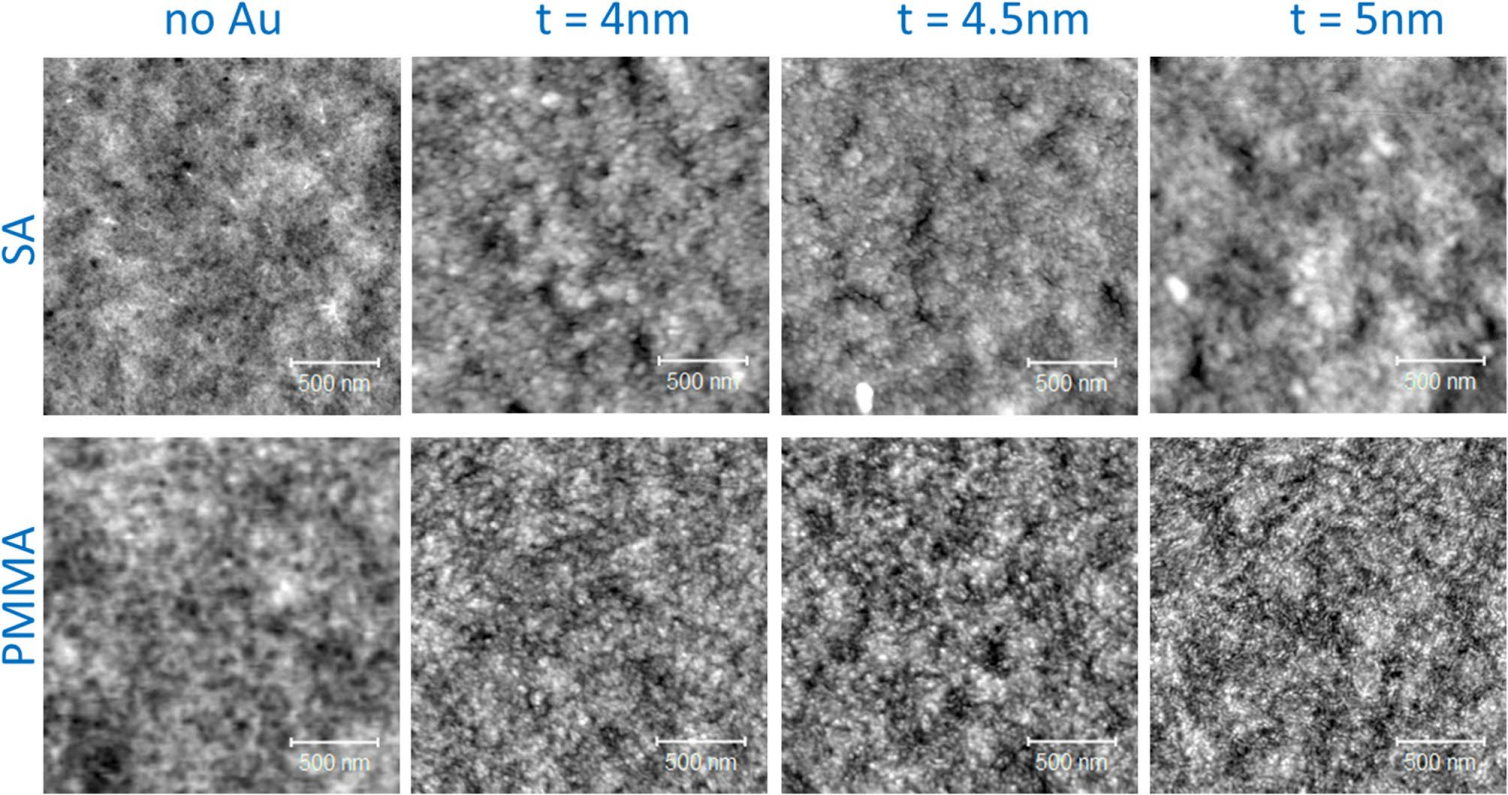
Atomic force microscopy analysis. Morphological data of the Au films sputtered on SA (upper panels) and PMMA (lower panels) films supported on glass.

The morphological analysis show that both the SA and PMMA films are very flat (Fig. 9), with a RMS roughness of 0.3 and 0.2 nm, respectively. The SA films compared to the PMMA ones present a different texture with smaller features. All the Au films are also very flat, though rougher than the pristine films. The Au films on PMMA compared to SA present smaller grainy texture. The RMS values increase with increasing the Au thickness, with a maximum RMS roughness of 0.5 nm on SA and 0.6 nm on PMMA for an Au thickness of 5 nm. These data do not evidence a clear morphological transition of the Au layer in a thickness range from 4 to 5 nm. In a previous study carried out on SA foils^15^, it has been shown that the Au clusters observable on the surface lie on top of other clusters embedded in the polymeric matrix. The transition in the electrical conductance can be thus attributed to reaching a critical proximity of the clusters embedded in the polymeric matrix.

### Electrical transport and noise measurements

The electric transport characterizations of the samples under test were performed in a temperature stabilized closed-cycle refrigerator, mod. Janis CCS-350S (Lake Shore Cryotronics, Westerville, OH, USA), covering a wide range of temperature from 300 to 10 K. Low noise DC and AC electronic bias and readout were used. In particular, the applied bias current was supplied with a DC current source, mod. Keithley 220 (Tektronix, Beaverton, OR, USA). While, the AC signal was amplified with a home-made electronics, optimized for low-noise measurements^39–41^, and was acquired by a dynamic signal analyzer type HP35670A (Keysight Technologies, Santa Rosa, CA, USA).

**Figure 10:**
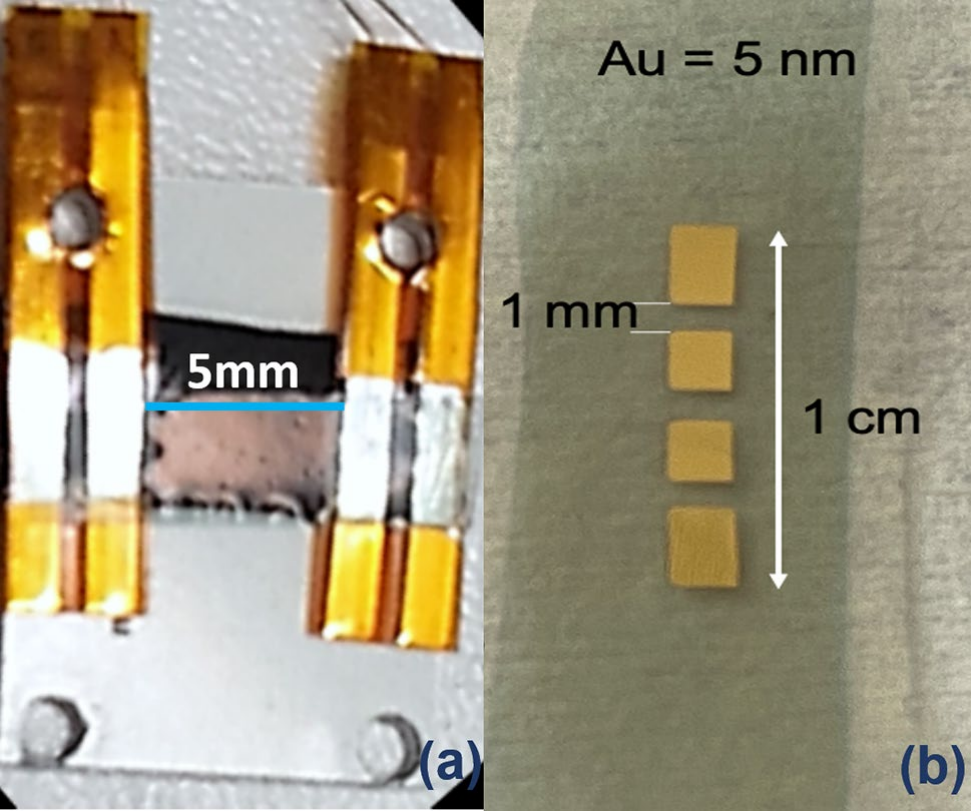
Electrical contacts configuration. (**a**) Photograph of the sample holder used for measurements realized with mechanically pressed contact pads. (**b**) Photograph of a typical investigated sample with inline Au pads deposited onto the surface for the electrical connections.

Here, it is important to underline the fact that the background curves contain the relevant information and, therefore, the peaks visible in the voltage-noise spectra are not considered in the analysis, as they are due to external spurious sources. These unwanted noise contributions can also be generated by the contacts. In order to reduce such extrinsic components, two different types of electrical connections were made. The first one used two Au covered copper strips embedded in Kapton, placed at a distance of about 5 mm, mechanically pressed onto the sample surface to obtain a four contacts geometry (see Fig. 10a, for details). Alternatively, a second type of contact configuration was realized by depositing four Au pads, 60 nm-thick and 1 mm-distant from each other, connected to the wires with silver paste (see Fig. 10b, for details). In all the cases, the contact noise was found to be negligible compared to the overall electric noise measured.

## Supplementary Information

Supplementary Information.

## Data Availability

The data that support the findings of this study are available from the corresponding authors upon reasonable request.
