# Supplementary material for: Electrical conduction and noise spectroscopy of sodium-alginate gold-covered ultrathin films for flexible green electronics
Source: Sci Rep. 2022 Jun 14;12:9861. doi: 10.1038/s41598-022-14030-2 (PMC9198047; doi:10.1038/s41598-022-14030-2)
Supplement: Supplementary file 1 — Supplementary Information. [file 41598_2022_14030_MOESM1_ESM.pdf]

Supplementary Information:

Electrical conduction and noise spectroscopy of sodium-alginate gold-covered ultrathin films for flexible green electronics

Carlo Barone*[,1,2,3], Piera Maccagnani[4], Franco Dinelli[5], Monica Bertoldo[6,7], Raffaella Capelli[8,9,10], Massimo Cocchi[7], Mirko Seri[11], and Sergio Pagano[#,1,2,3]

[1] *Dipartimento di Fisica "E.R. Caianiello", Università degli Studi di Salerno, Via Giovanni Paolo II 132, 84084 Fisciano (SA), Italy*

[2] *CNR-SPIN Salerno, c/o Università degli Studi di Salerno, 84084 Fisciano (SA), Italy*

[3] *INFN Gruppo Collegato di Salerno, c/o Università degli Studi di Salerno, 84084 Fisciano (SA), Italy*

[4] *CNR-Istituto per la Microelettronica e Microsistemi, Via P. Gobetti 101, 40129 Bologna, Italy*

[5] *CNR-Istituto Nazionale di Ottica, Via G. Moruzzi 1, 56124 Pisa, Italy*

[6] *Dipartimento di Scienze Chimiche, Farmaceutiche ed Agrarie, Università degli Studi di Ferrara, Via L. Borsari 46, 44121 Ferrara, Italy*

[7] *Istituto per la Sintesi Organica e la Fotoreattività, Consiglio Nazionale delle Ricerche, Via P. Gobetti 101, 40129 Bologna, Italy*

[8] *Dipartimento di Ingegneria E. Ferrari, Università di Modena e Reggio Emilia, 41125 Modena, Italy*

[9] *CNR-Istituto Officina dei Materiali, S.S. 14, km 163.5 in Area Science Park, 34012 Trieste, Italy*

[10] *Department of Physics, University of Johannesburg, P.O. Box 524, Auckland Park 2006, South Africa*

[11] *CNR-Istituto per lo Studio dei Materiali Nanostrutturati (ISMN), Via Piero Gobetti 101, 40129 Bologna, Italy*

E-mail: cbarone@unisa.it or spagano@unisa.it

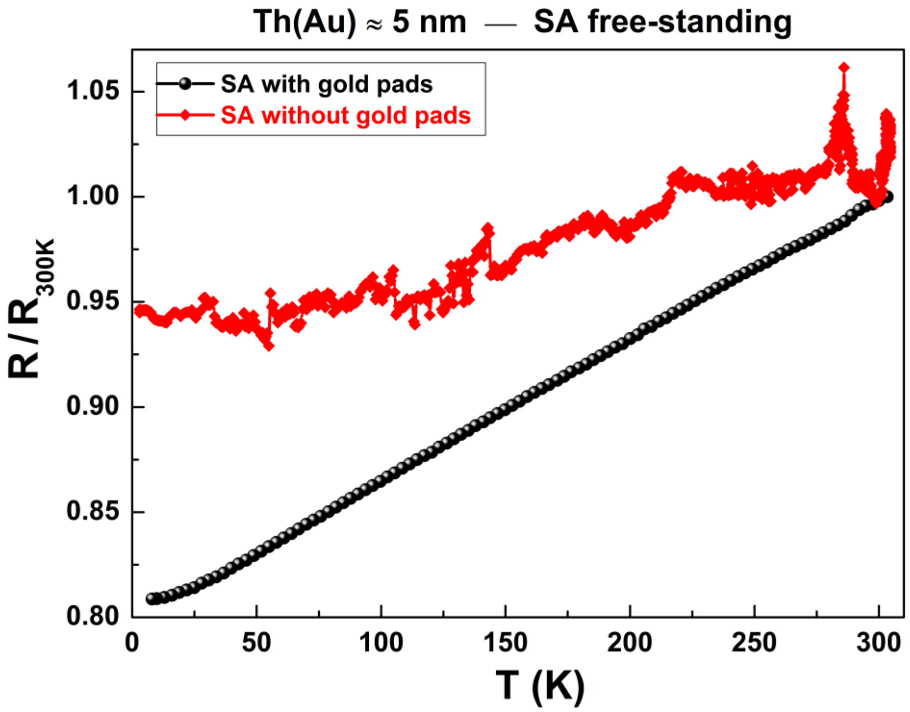

Supplementary Figure 1 | Normalized resistance versus temperature plots of metallic films

The temperature dependence of the normalized resistance R/R_{300K} is shown for SA free-standing films covered with a 5 nm-thick Au layer. The results are reported for different types of electrical connections: with Au pads (black circles) or without Au pads (red diamonds).
